# Patients at high risk for a severe clinical course of COVID-19 — small-area data in support of vaccination and other population-based interventions in Germany

**DOI:** 10.1186/s12889-021-11735-3

**Published:** 2021-09-28

**Authors:** Jakob Holstiege, Manas K. Akmatov, Claudia Kohring, Lotte Dammertz, Frank Ng, Thomas Czihal, Dominik von Stillfried, Jörg Bätzing

**Affiliations:** Central Research Institute of Ambulatory Health Care, Salzufer 8, 10587 Berlin, Germany

**Keywords:** COVID-19, Germany, Patients at risk, SARS-CoV-2, Vaccination, Vulnerable population

## Abstract

**Background:**

Research has shown that the risk for a severe course of COVID-19 is increased in the elderly population and among patients with chronic conditions. The aim of this study was to provide estimates of the size of vulnerable populations at high risk for a severe COVID-19 course in Germany based on the currently available risk factor data.

**Methods:**

We used nationwide outpatient claims data from the years 2010 to 2019 collected according to § 295 of the Code of Social Law V, covering data for all statutory health insurees (SHI) which is nearly 87% of the entire German population. We considered 15 chronic disorders based on the current state of knowledge about clinically relevant risk factors. Three risk groups for a severe COVID-19 course were defined: 1. individuals in the age group 15 to 59 years with at least two comorbid disorders; 2. individuals aged 60 to 79 years with at least one disorder and 3. all individuals 80 years and older irrespective of the presence of chronic conditions. Regional analysis was conducted at the level of administrative districts (*n* = 401).

**Results:**

Overall, 26% of individuals over 15 years were at high risk for a severe COVID-19 course in 2019 amounting to a total number of nearly 18.5 million individuals in Germany. This included 3.8 million individuals in risk group 1, 9.2 million in risk group 2, and 5.4 million in risk group 3, corresponding to 8, 50 and 100% of German inhabitants in the respective age groups. On the level of the 17 administrative regions formed by the Association of SHI Physicians (ASHIP regions), the proportion of individuals at high risk ranged between 21% in Hamburg and 35% in Saxony-Anhalt. Small-area estimates varied between 18% in Freiburg (Baden-Württemberg) and 39% in the district Elbe-Elster (Brandenburg).

**Conclusions:**

The present study provides small-area estimates of populations at high risk for a severe COVID-19 course. These data are of particular importance for planning of preventive measures such as vaccination.

**Trial registration:**

not applicable.

**Supplementary Information:**

The online version contains supplementary material available at 10.1186/s12889-021-11735-3.

## Background

The proportion of the population aged older than 70 years in Germany is one of the highest in Europe and, in combination with a prevalence of chronic conditions, leads to an increased vulnerability for severe health impairments due to COVID-19 [1]. In May 2020, we made available the case numbers of patients with an increased risk of an unfavorable course of COVID-19 on the district level based on the state of knowledge at the end of March 2020 [2]. Five disease groups including hypertension, heart failure, type 1 and 2 diabetes mellitus, chronic obstructive pulmonary disease (COPD), as well as congenital and acquired diseases of the immune system were considered for this purpose. Meanwhile, further risk factors for a severe clinical course of COVID-19 have been identified [3–6].

First recommendations for the prioritization of risk groups were already published in a position paper by the German Standing Committee on Vaccination (STIKO) in cooperation with the German National Academy of Sciences Leopoldina and the German ethics council back in November 2020 [7]. Among others, persons who *‘[ …*] *due to their age or already impaired health conditions have a significantly increased risk of a severe or fatal disease course [ …*]’ should be prioritized for vaccination [7]. The first information about risk groups with specific chronic conditions was published in a position paper from 23. November 2020 by the World Health Organization (WHO) [8, 9]. Among others, patients with cancer, diabetes mellitus, chronic diseases of heart, liver, lungs or kidneys, neurological and immunological diseases including organ transplantations as well as individuals with obesity should be prioritized for vaccination. This selection of chronic conditions was supported by several other studies [5] and reviews [4, 6]. Current clinical data from the Italian Istituto Superiore di Sanitá confirm an almost identical profile [10].

The Federal Joint Committee (Gemeinsamer Bundesausschuss; G-BA) has recently estimated case numbers of vulnerable individuals in Germany based on a systematic literature review [11]. The aim was to assess the need of FFP-2 masks for the planned distribution to at-risk individuals. All persons aged 60 years or older irrespective of the presence of chronic diseases as well as persons under 60 years with selected chronic conditions were included. Overall, the vulnerable population comprised 27.2 million people in Germany [11]. This estimation is in close agreement with the result of our recent study on the amount of individuals with chronic diseases for whom a vaccination against influenza is recommended by the STIKO [12]. Generally, there is a high degree of overlap between the indications for vaccination against influenza and COVID-19. The afore-mentioned analysis investigated more than 70 diagnoses and thereby included nearly all individuals aged 60 years and older. According to our study, 28.3 million SHI individuals had at least one chronic condition and were thereby part of the group with a disease-related indication for an influenza vaccination.

Data on the size of populations under high risk for a severe disease course of COVID-19 are of primary importance for the planning of the vaccination strategy against COVID-19. Considering the limited availability of vaccines against COVID-19, especially in the early phase of the vaccination period in Germany, prioritisation of relevant risk groups was essential. The present study aimed at regionally assessing the size of vulnerable populations at high risk for a severe COVID-19 course on district level based on the current state of knowledge. Overall, 15 chronic conditions or disease groups were considered in the analysis.

## Methods

### Database and study population

The study was based on nationwide pseudonymized outpatient claims data encompassing all German statutory health insurances from the years 2010 to 2019 collected in accordance with §295 of the Code of Social Law V (Sozialgesetzbuch, SGB V) [13]. This database is a comprehensive collection of administrative outpatient data of all SHI individuals (87% of the entire German population). Besides sociodemographic characteristics such as age, sex and place of residence, the data include, amongst others, information on billed medical services and diagnoses as well as physician-related characteristics such as specialist group and practice location. Diagnoses are coded according to the International Statistical Classification of Diseases and Related Health Problems, 10th revision, German modification (ICD-10-GM). In addition, diagnoses from German outpatient care include a modifier describing the diagnostic certainty (‘assured’, ‘suspected’, ‘status post’, ‘excluded’) [14]. The study population included individuals aged ≥15 years (*N* = 61,533,884).

### Selection of prognostically relevant pre-existing conditions

The selection of pre-existing conditions was based on previous findings on risk factors for severe course of COVID-19 disease related to chronic health conditions reported in international studies. We included chronic conditions that were associated with a markedly increased age-adjusted risk in several epidemiological studies of COVID-19 patient groups or the general population based on selective literature search. Underlying chronic conditions for which there was no clear evidence in the selected literature were not considered. A markedly increased risk was generally assumed, if the risk of the outcomes hospitalization, intensive care, and/or death was increased by the factor 2 or more in age-adjusted analysis in at least one of the identified studies. The prognostically relevant chronic conditions included for the definition of high risk groups are given in Table 1.

**Table 1:** Chronic conditions included in the study with the corresponding ICD-10 codes

Diseases / disease groups [source]	ICD-10 codes
Obesity [15]	E66
Chronic obstructive pulmonary disease, COPD [16]	J44
Chronic kidney disease / renal failure [17]	N18, N19
Chronic liver disease [18]	B18, K70, K72.1, K73, K74
Dementia [5]	F00–F03
Type 2 diabetes mellitus [19]	E11–E14
Disease-related immunosuppression (except HIV infection, tumor diseases und drug related immunosuppression (if the last two disease groups were not coded by D90) [20]	D73.0, D80.-, D81.-, D82.-, D83.-, D84.-, D86.-, D90
Arterial hypertension [5]	I10–I15
Cardiovascular diseases [21, 22]	
Coronary heart disease	I20–I25
Heart failure	I50, I11.0, I13.0, I13.2
Solid tumors [5]	C00–C80, without C44
Hematological tumors [23]	C81–C96
Stroke or post-stroke condition and cerebrovascular precursors [24]	I63–I66
Other neurological diseases [5]	
Multiple sclerosis	G35
Parkinson‘s disease	G20
Transplantations or post-transplantation conditions of kidney, lung, heart, heart-lung or liver [25, 26]	Z94.0–Z.94.4

### Assessment of chronic conditions

In the population of all SHI individuals in the year 2019, occurrence of the included chronic conditions was assessed on the individual level. For the majority of diseases or disease groups this was done by using the so called M2Q-criterion. Accordingly, patients were defined as having prevalent chronic diseases, if they had a diagnosis coded with the diagnostic certainty ‘assured’ for the respective condition or disease group in at least two quarters of the year 2019. For the identification of cases per disease entity according to the M2Q-criterion, it was irrelevant if identical or different diagnoses from the list of ICD-codes per disease group were assigned in at least two quarters. For disease groups with assigned subgroups (cardiovascular diseases and other neurological diseases) the M2Q-criterion had to be met for at least one of the respective subgroups.

For solid tumors, hematological tumors, stroke or post-stroke conditions, cerebrovascular precursors as well as transplantations and post-transplantation conditions the case definition was modified. For the assessment of stroke or post-stroke conditions and cerebrovascular precursors according to the M2Q-criterion, diagnoses coded with the diagnostic certainty ‘status post’ were considered in addition to ‘assured’ diagnoses. The same approach was applied for transplantations and post-transplantation conditions. In contrast, solid tumors were included as incident diseases, if the M2Q-criterion applied for at least one of the considered ICD-three-character-codes in 2018 and/or 2019 and if there was no diagnosis coded with diagnostic certainty ‘assured’ of a solid tumor in the years 2010 to 2017. Current evidence suggests an especially increased risk associated with hematological tumors [23], even with a first occurrence of the disease in the past [5]. Hence, patients who met the M2Q-criterion at least once for one of the considered ICD-three-character-codes in the years 2015 to 2019 were also included.

### Classification of vulnerable populations at high risk for a severe COVID-19 course

The continuously growing study base indicates the high relevance of age and specific chronic conditions for the prognosis of severe COVID-19 courses. Early after the onset of the pandemic, a higher age emerged as the most important predictor for the need of intensive care due to COVID-19 [27, 28]. Furthermore, epidemiological studies showed a largely consistent pattern of a markedly increased risk associated with the chronic conditions included in this study. However, only limited evidence is available on the interaction of age and chronic conditions as well as chronic conditions with each other. The majority of the reviewed epidemiological studies and meta-analyses did not consider the interaction of age and chronic conditions in the statistical models applied for the risk estimation. Regarding the interplay of several comorbidities, individual studies confirm a clinically plausible accumulation of risks from single chronic conditions [29, 30].

Under the pragmatic assumption of essentially aligned additive associations of age and comorbidity related risk, a high risk of severe COVID-19 courses was postulated for the following groups and classified respectively:

- Risk group 1: People aged 15 to 59 years with two or more prognostically relevant chronic conditions or a hematological tumor or a specific transplantation or an immunosuppressive disease
- Risk group 2: People aged 60 to 79 years with at least one prognostically relevant chronic condition
- Risk group 3: People aged 80 years and older irrespective of underlying chronic conditions

Based on the profile of the included chronic conditions on the individual patient level, we calculated the prevalence of at least two prognostic relevant chronic conditions for the age group 15 to 59 years and of at least one for the age group of 60 to 79 years on district level. As analyses on interacting effects of the risk factors hypertension and age suggested a decreasing risk of hypertension with increasing age in a large British population-based study [5], hypertension was not classified as a chronic condition with high prognostic relevance in the age group 60 to 79 years.

In addition to the assessment of population sizes of the three predefined risk groups, we calculated the total number of individuals with at least one of the relevant chronic conditions in the age group 15 to 59 years. To assess robustness of our results based on morbidity profiles captured from SHI claims and population statistics of 2019, analyses were repeated using 2018 data.

### Small-area estimation of vulnerable populations

The estimation of the regional populations’ size among German inhabitants with a high risk for a severe COVID-19 course was conducted for the three risk groups. The risk groups 1 and 2 were created based on an extrapolation of the respective population-based prevalence of risk group in the SHI population to the population of all German inhabitants in the respective age group. A pragmatic fundamental assumption was that the regional age-specific morbidity in the SHI population was similar to that of the general population. The risk group 3 included the complete population aged 80 years and older irrespective of the presence of chronic conditions. The extrapolation was conducted using population data on administrative district level from the German regional database of the federal and state statistical offices on www.regionalstatistik.de.

## Results

On the national level, an overall number of about 18.5 million people showed a high risk for a severe course of COVID-19. The percentage of this vulnerable group in the general population aged ≥15 years was 26%. Nationwide, the proportion in those aged 15 to 59 years at high risk amounted to 8%, and for those aged 60 to 79 years to 50%, while all inhabitants in the age group 80 years and older (i.e. 100%) were considered to exhibit a high risk (Table 2).

**Table 2:** Absolute number of individuals and percentage of the population with a high risk for a severe course of COVID-19 (high risk groups) by region of Association of Statutory Health Insurance Physicians (ASHIP region) stratified by three age groups (15 to 59 years, 60 to 79 years and ≥ 80 years) based on nationwide outpatient claims data and population statistics of German inhabitants from 2019

ASHIP region or federal state	15–59 years		60–79 years		≥80 years		Total	
	Population at risk [n]	Risk prevalence [%]	Population at risk [n]	Risk prevalence [%]	Population at risk [n]	Risk prevalence [%]	Population at risk [n]	Risk prevalence [%]
Baden-Württemberg	400,085	6.1	1,028,252	44.8	688,972	100.0	2,117,309	22.2
Bavaria	545,887	7.0	1,357,978	49.2	792,240	100.0	2,696,105	23.8
Berlin	158,785	7.1	379,998	53.8	203,150	100.0	741,933	23.6
Brandenburg	147,432	10.9	367,572	57.0	187,053	100.0	702,057	32.1
Bremen	30,633	7.6	71,578	50.3	43,847	100.0	146,058	24.8
Hamburg	71,472	6.2	159,410	48.3	104,060	100.0	334,942	21.2
Hesse	281,862	7.6	661,319	49.8	387,621	100.0	1,330,802	24.6
Mecklenburg-Western Pomerania	102,675	11.9	238,029	56.4	119,703	100.0	460,407	32.8
Lower Saxony	400,142	8.7	906,898	50.9	529,905	100.0	1,836,945	26.6
North Rhine*	454,149	8.0	1,040,672	50.5	622,280	100.0	2,117,101	25.4
Rhineland-Palatinate	199,309	8.5	470,792	50.8	269,801	100.0	939,902	26.5
Saarland	48,684	8.9	126,503	51.8	72,445	100.0	247,632	28.6
Saxony	194,707	9.0	564,017	54.6	335,427	100.0	1,094,151	31.0
Saxony-Anhalt	143,263	12.4	349,298	58.7	173,394	100.0	665,955	34.6
Schleswig-Holstein	134,430	8.2	323,685	48.4	197,663	100.0	655,778	26.1
Thuringia	123,102	10.9	322,547	56.6	160,543	100.0	606,192	32.6
Westphalia-Lippe*	386,046	8,1	873,593	48.8	544,704	100.0	1,804,343	25.4
Germany	3,822,663	8.0	9,242,141	50.5	5,432,808	100.0	18,497,612	25.8
North Rhine-Westphalia*	840,195	8.1	1,914,265	49.7	1,166,984	100.0	3,921,444	25.4

* The two ASHIP regions, North Rhine und Westphalia-Lippe are additionally shown summarized as the federal state North Rhine-Westphalia

By including all people aged 15 to 59 years with at least one of the relevant chronic conditions in the high risk group, the absolute number of people with a particularly high need of protection increases by about 34% to an overall amount of 24.8 million.

**Fig. 1:**
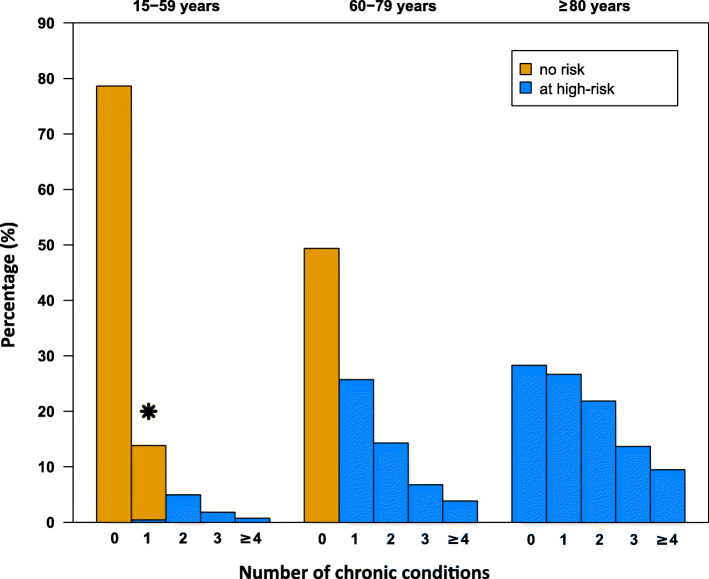
Percentages of patients by number of relevant chronic conditions with a high risk for a severe course of COVID-19 in the age groups 15 to 59 years, 60 to 79 years and 80 years. Orange bars show the percentage of patients without risk, blue bars the percentage of patients at high risk. *This bar includes a small proportion of 0.4% of patients at high risk with only one chronic condition (hematological tumor, transplantation, or immunosuppressive condition)

Figure 1 shows the risk prevalence of the three age groups in the SHI population, if stratified by the number of relevant chronic conditions. Overall, 79, 49 and 28% in the age groups 15–59 years, 60–79 years and ≥ 80 years exhibited none of the chronic conditions considered for risk classification, respectively (Fig. 1). Prevalence estimates of single disease groups in the age groups 15 to 59 years and 60 to 79 years in 2019 are depicted in supplementary Table S1.

Regional distinction according to areas covered by the 17 Associations of Statutory Health Insurance Physicians (ASHIP regions) in Germany shows a percentage of persons at high risk ranging from 21% in Hamburg to 35% in Saxony-Anhalt (Table 2). Supplementary Fig. S1 shows the absolute numbers of the high risk population differentiated between districts of eastern and western federal states.

**Fig. 2:**
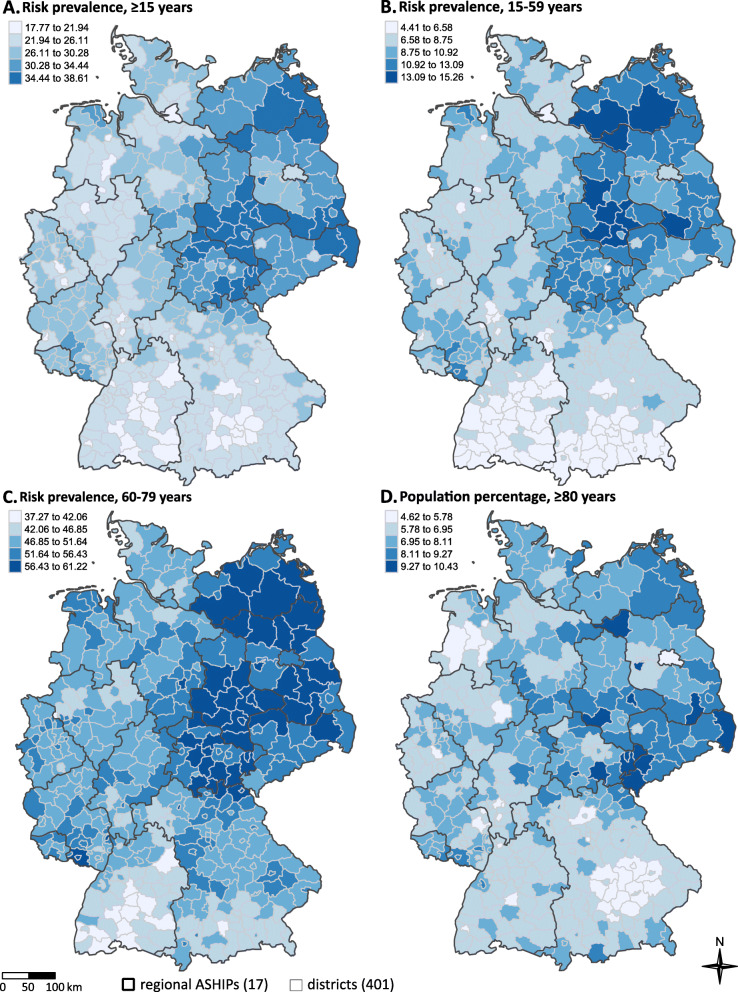
Percentage of the total population aged ≥15 years (A.), the age group 15–59 years (B.) and the age group 60–79 years (C.) at high risk for a severe course of COVID-19 (high risk group) as well as the percentage of German inhabitants aged ≥80 years (D.) on district level (*n* = 401 districts) based on nationwide outpatient claims data and population statistics of German inhabitants from 2019

On the level of administrative districts and over all age groups, the proportion of individuals with a high risk ranged from 18% in the urban district of Freiburg (Baden-Württemberg) to 39% in the district Elbe-Elster (Brandenburg, Fig. 2A). In the age groups 15–59 years and 60–79 years the prevalence of high risk varied between 4.4% (Starnberg, Brandenburg) and 15.3% (Elbe-Elster, Brandenburg, Fig. 2B) and 37.23 (Reutlingen, Baden-Württemberg) and 61.2% (Prignitz, Brandenburg, Fig. 2C), respectively.

The percentage of the general population at high risk for a severe course of COVID-19 based on insurance claims and population statistics of the year 2018 was estimated to amount to 25.4% (2019: 25.8%), 7.9% (2019: 8.0%) and 50.3% (2019: 50.5%) in German inhabitants aged ≥15 years, 15–59 years and 60–79 years, respectively.

## Discussion

The present study yields small-area estimates of the population size with a high risk for a severe COVID-19 course; it is based on current evidence from a continuously growing body of international epidemiological studies. Given temporarily limited resources for vaccination of the population, this study presents an empirical basis for the assessment of regionally differing demands and for the planning of prioritized vaccine allocations depending on chronic disease profiles and the age structure of the population. The results reveal strong regional differences and particularly high values in eastern German districts regarding the proportion of people for whom a high risk can be assumed.

In May 2020, we published the estimated case numbers of patients with an increased risk for an unfavorable course of COVID-19 on district level based on the state of knowledge by March 2020, which were initially used to support the Associations of Statutory Health Insurance Physicians to support planning of allocation of health services during the COVID-19 pandemic [2]. Since then, a variety of new findings on pre-existing chronic conditions associated with a high risk of severe COVID-19 courses has been published. As already suspected in our first publication in May, a significant role of pronounced obesity has meanwhile been confirmed [15]. Moreover, patients with pre-existing cancers by now have to be attributed to the vulnerable population, however, in different extents for hematological and solid tumors [5, 23]. Hypertension was shown to have a negative impact especially in younger age [5]. Further chronic conditions such as chronic kidney disease and renal failure [17], chronic liver diseases [18], dementia [5] as well as stroke and other neurological conditions [5, 24] were added as relevant prognostic conditions associated with an increased risk for a severe disease course of COVID-19. Also, asplenia and post-transplantation conditions are associated with a higher risk [5, 25, 26]. Advanced age, however, was identified as the most relevant predictor for intensive care and death due to COVID-19, independent from underlying chronic conditions [28, 29, 31].

We took these risk assessments into account by specifically assigning the proportions of highly vulnerable populations to different age groups. This pragmatic, but compared to our previous analysis from March 2020, more restrictive approach, resulted in a number of about 18.5 million people with a high risk for a severe course of COVID-19 when extrapolating outpatient claims data from the SHI-insured population to the general population. The proportion of these highly vulnerable individuals in the general population aged ≥15 years amounts to 26% on the national level, and it ranges between 21% in the region of Hamburg and 35% in the Saxony-Anhalt region.

In agreement with prior expectations, data on the level of German states and districts revealed clustering of elevated prevalence estimates of increased vulnerability in the age groups 15–59 years and 60–79 years in East Germany. In addition, the percentage of the population aged ≥80 years was higher in a majority of eastern in contrast to western German districts. Following the German unification in 1990 young eastern residents disproportionately migrated to economically strong western regions, resulting in accelerated population ageing in East Germany [32]. Furthermore, even when adjusted for regional variations in populations’ age structures eastern German residents are more likely to be affected by wide spread chronic conditions including cardiovascular risk factors and diseases [33–35] and chronic obstructive pulmonary disease [36]. Some of the variations of disease burden between East and West Germany can be explained by differences of socioeconomic conditions between regions [35]. Undoubtedly, the size of the population with at least one of the considered chronic conditions associated with a severe disease course of COVID-19 exceeds that of the group with a high risk quantified in this study. By including all people aged 15 to 59 years with at least one of the relevant chronic conditions in the high risk group, the absolute number of people with a particularly high need of protection increases by about 34% to an overall amount of 24.8 million.

Back in November 2020, in the context of prospectively planning the COVID-19 vaccinations, the position paper from the STIKO, the German National Academy of Sciences Leopoldina and the German Ethics Council already recommended among others that *‘[ …] persons (person groups), who have a significantly higher risk for a severe or fatal course due to their age or pre-existing health impairments, especially when exposed to an increased density of contacts (such as nursing homes and other long-term care facilities)[ …]’* should be vaccinated with priority [31]. This largely corresponded to the WHO recommendations, which for the early stage of vaccine availability with initial temporary shortage, suggest vaccination for people of a certain age (e.g. from the age of 60 years) and for those aged younger, when affected by specific pre-existing conditions [8, 9].

Our data aimed to support the planning of the prioritization of vaccination on the patients’ side for highly vulnerable groups based on empirical data. Our analyses identified the needs that can be expected for priority vaccination of highly vulnerable groups down to the district level.

Pregnant women, for whom an increased risk has by now also been reported especially for those with older age, higher body weight and underlying diseases, were intentionally not included in the analysis [37]. Also, the WHO is critical of a prioritization for this group due to insufficient experiences with the vaccines [9]. From the authors’ point of view this similarly holds true for vaccination priorities for children and adolescents, who, however, have a generally very low risk of a severe COVID-19 course [38] and, therefore, no imperative need for prioritized vaccination. At the time of analysis, none of the available vaccines had been approved for administration to children and adolescents under the age of 16. Confirmatory indications from the STIKO suggested that children and adolescents up to the age of 15, and pregnant women will probably not be considered for vaccination at this early stage of the national vaccination campaign. Therefore, we did not consider pregnancy in the present study and defined a minimum age of 15 years as an inclusion criterion.

A very high risk has by now also been shown for some rare diseases, such as e.g. a 25-fold increased risk of death with COVID-19 for patients with Down’s syndrome compared to age- and sex-matched controls [39]. In all of these constellations, the decision about vaccination needs to be the result of a careful individual risk-benefit assessment by a specialist against the background of yet insufficient data about vaccine tolerability.

Generally, the results of the present study may also be helpful for the detailed planning of small-area allocation of other measures to reduce viral transmission, such as the distribution of FFP-2 masks to patients with an increased COVID-19 risk. To date, the G-BA recommends the age of 60 years and older as an inclusion criterion irrespective of relevant chronic conditions [11].

Inpatient accommodation in sheltered establishments as well as in nursing or old people’s homes is associated with an increased risk of exposure to SARS-CoV-2 and confers a clearly increased risk for a severe and fatal course of COVID-19 because of the raised morbidity due to relevant chronic conditions [40]. Properties of outpatient claims that allow to identify nursing home residents are limited with regard to their sensitivity [41]. Yet, in a sub-population of 154,489 patients aged 70 to 79 years, who could clearly be assigned to this group based on outpatient physician claims, 132,397 patients (86%) were identified by the disease algorithm as high risk patients. Due to inclusion of all people aged 80 years and older into the population at high risk, we estimate the proportion of residents of nursing or old people’s home, who were included in the high risk group to be higher than 97%. Therefore, it can be assumed that the capture of people accommodated in nursing or old people’s facilities through the classification of risk groups based on selected pre-existing chronic conditions with high COVID-19 risks was nearly complete.

### Strengths and limitations

A specific strength of our research results from the coverage of insured people encompassing all SHI individuals and the supra-regional character of the outpatient claims data used. Due to a high frequency of comorbid occurrence of included relevant chronic conditions for risk classification, the use of results from previous primary data studies with usually a focus on single diseases comes with the risk to overestimate the size of the vulnerable population. At the same time, the assessment of the combined occurrence of single risk factors from the entire spectrum of the relevant chronic conditions allows the description of accumulated risks on the level of individual patients.

Some limitations have to be mentioned. The data set of the present study did not capture chronic conditions associated with a severe COVID-19 course that have been diagnosed in inpatient settings only. However, sole inpatient treatment is assumed to be relatively rare. Furthermore, patterns of distribution of morbidity in the SHI population have been extrapolated to the general German population, which in 2019 also comprised about 11% of inhabitants covered by private insurance. Due to an assumed somewhat lower prevalence of relevant risk factors in the privately insured population, which has been shown for some chronic conditions, our findings may have overestimated the size of the population with high vulnerability to an unknown but at most moderate extent. In the used SHI claims data, pseudonymized patient identifiers are formed by assigning unique integers to every distinct combination of a patient’s first and last name and a patient’s date of birth. A patient’s pseudonym can change permanently in case of name changes (e.g. by marriage). Furthermore, a patient’s pseudonym may temporarily change due to occasionally occurring erroneous data entries in outpatient physicians’ offices. As a result with regard to prevalence estimates both numerator (outpatients aged ≥15 years with specific diagnoses in two quarters of 2019) and denominator (all outpatients aged ≥15 years) likely were subject to double counting of a minority of patients with changes of attributes used to form patients‘pseudonyms during 2019. As both numerator and denominator are simultaneously affected to a similar extent, it can be assumed that prevalence estimates closely reflect the true morbidity in the SHI population.

The current study aimed to give an estimate of the size of populations with a high risk of a severe COVID-19 course to allow appraisal of regionally varying needs for vaccination due to morbidity differences from pre-existing chronic conditions. Due to a lack of detailed clinical data in insurance claims the employed algorithm features limited capabilities to differentiate between subgroups of patients depending on the severity and course of chronic conditions and hence may result in misclassification of individual risks in some patients. In case of solid cancers the population at increased risk was likely underestimated since only patients with incident disease in 2018 or 2019 were captured, but patients with cancer relapse in 2018 or 2019, who were already treated for cancer sometime during the preceding seven year period were excluded from case definition.

Since only limited evidence exists on the interaction of risks of age and chronic conditions as well as chronic conditions with each other, risk groups were classified using a pragmatic approach. Accordingly, essentially aligned additive associations of age and comorbidity related risk were assumed. This approach allowed to give estimates on the size of populations with high vulnerability incorporating cumulation of individual risks. As the nature of interactions of comorbidities is unknown, this approach likely oversimplyfied the interplay of single risk factors, which may be rather mutiplicative than additive in many cases.

## Conclusions

The present study provides small-area estimates of the size of vulnerable populations for a severe COVID-19 course by using a pragmatic approach with regard to the classification of high risk, pending the cumulation of risk by age and number of chronic conditions. Strong regional differences and especially high values in eastern German districts regarding the proportion of high risk populations may inform targeted regional planning of preventive measures such as vaccination. In future pandemic situations, rapid assessment of regional sizes of populations under increased risk should be the basis for regional distribution of vaccines in an early phase of limited vaccine availability.

## Supplementary Information

**Additional file 1. Table S1.**

**Additional file 2. Figure S1.**

## Data Availability

The datasets analyzed during the current study are not publicly available due to data protection regulations by the Code of Social Law V (Sozialgesetzbuch, SGB V).

## Abbreviations

COPD: chronic obstructive pulmonary disease
COVID: coronavirus disease
G-BA: Federal Joint Committee
ICD-10: International Statistical Classification of Diseases and related Health Problems, 10th revision
SGB: Code of Social Law
SHI: statutory health insurance
STIKO: German Standing Committee on Vaccination
WHO: World Health Organization
