# Supplementary material for: Patients at high risk for a severe clinical course of COVID-19 — small-area data in support of vaccination and other population-based interventions in Germany
Source: BMC Public Health. 2021 Sep 28;21:1769. doi: 10.1186/s12889-021-11735-3 (PMC8478008; doi:10.1186/s12889-021-11735-3)
Supplement: Supplementary file 1 — Additional file 1. Table S1. [file 12889_2021_11735_MOESM1_ESM.docx]

**Table S1 (online supplement).** Prevalence of single disease groups used for the classification of vulnerable populations at high risk for a severe COVID-19 based on cumulative burden of morbidity in the age groups 15 to 59 years and 60 to 79 years in 2019

| **Disease groups** | **Age group 15-59 years (n=40.476.182)** | | **Age group 60-79 years (n=15.392.486)** | |
| --- | --- | --- | --- | --- |
|  | **patients** | **prevalence (%)** | **patients** | **prevalence (%)** |
| Obesity | 2,808,131 | 6.94 | 2,343,211 | 15.22 |
| Chronic obstructive pulmonary disease, COPD | 689,734 | 1.70 | 1,421,217 | 9.23 |
| Chronic kidney disease / renal failure | 360,060 | 0.89 | 1,315,884 | 8.55 |
| Chronic liver disease | 243,079 | 0.60 | 236,402 | 1.54 |
| Dementia | 31,008 | 0.08 | 360,197 | 2.34 |
| Type 2 diabetes mellitus | 1,548,044 | 3.82 | 3,587,334 | 23.31 |
| Disease-related immunosuppression* | 205,174 | 0.51 | 123,503 | 0.80 |
| Arterial hypertension** | 5,668,012 | 14.00 | 9,159,429 | 59.51 |
| Cardiovascular diseases | 736,707 | 1.82 | 2,778,134 | 18.05 |
| Solid tumors | 232,545 | 0.57 | 390,212 | 2.54 |
| Hematological tumors | 119,133 | 0.29 | 194,465 | 1.26 |
| Stroke*** | 268,240 | 0.66 | 1,095,666 | 7.12 |
| Other neurological diseases | 194,591 | 0.48 | 210,493 | 1.37 |
| Transplantations**** | 26,892 | 0.07 | 21,206 | 0.14 |

*Disease-related immunosuppression except HIV infection, tumor diseases und drug related immunosuppression (if the last two disease groups were not coded by D90); **Hypertension was not classified as a chronic condition with high prognostic relevance in the age group 60 to 79 years (see methods). ***Stroke or post-stroke condition and cerebrovascular precursors; ****Transplantations or post-transplantation conditions of kidney, lung, heart, heart-lung or liver
