# Supplementary figures and images for: Patients at high risk for a severe clinical course of COVID-19 — small-area data in support of vaccination and other population-based interventions in Germany
Source: BMC Public Health. 2021 Sep 28;21:1769. doi: 10.1186/s12889-021-11735-3 (PMC8478008; doi:10.1186/s12889-021-11735-3)

Number of patients at risk (in thousand)

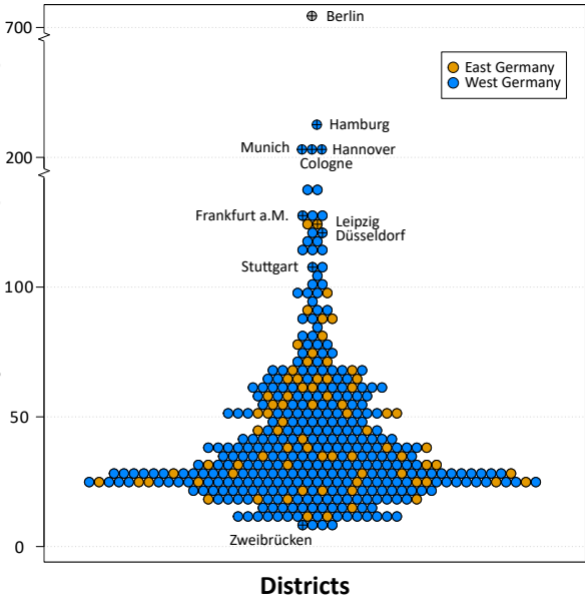

Supplement: Supplementary file 2 — Additional file 2. Figure S1. [file 12889_2021_11735_MOESM2_ESM.pdf]
